# Legal linguistic templates and the tension between legal knowledge representation and reasoning

**DOI:** 10.3389/frai.2023.1136263

**Published:** 2023-04-27

**Authors:** Tomer Libal

**Affiliations:** Department of Computer Science, University of Luxembourg, Luxembourg, Luxembourg

**Keywords:** automated reasoning, formal representation, knowledge validation, legal informatics, domain specific language, reverse translations

## Abstract

There is an inherent tension between knowledge representation and reasoning. For an optimal representation and validation, an expressive language should be used. For an optimal automated reasoning, a simple one is preferred. Which language should we choose for our legal knowledge representation if our goal is to apply automated legal reasoning? In this paper, we investigate the properties and requirements of each of these two applications. We suggest that by using Legal Linguistic Templates, one can solve the above tension in some practical situations.

## 1. Introduction

Information systems are playing an important role in helping people in a wide range of tasks, ranging from searching to decision-making.

One area in which such tools can contribute is the legal domain: New court cases and legislations are accumulated every day. In addition, international organizations like the European Union are constantly aiming at combining and integrating separate legal systems (Burley and Mattli, 1993).

Approaches for searching over legal texts have long seen commercial success (Weaver and Bimber, 2008). Other methods, for example for predicting legal outcomes (Aletras et al., 2016; Sulea et al., 2017), have been greatly discussed in the literature. At the same time, some tools for reasoning over sets of norms have been developed, such as for business (e.g., Hashmi and Governatori, 2018) and law (e.g., Palmirani and Governatori, 2018; Libal and Pascucci, 2019). Among the applications of such tools are legal drafting, consistency checks, and for deducing implications (Prakken and Sartor, 2015).

The most important application for these tools, though, is for capturing expertise. Expert systems (Waterman, 1985) have been successfully utilized in various domains, among them in finance (Connell, 1987) and health (Durkin, 1996).

Nevertheless and despite their proven usefulness, expert systems are not widely used in the legal domain. The main reason for that is the difficulty to capture the expert knowledge within a computer program (McGraw and Harbison-Briggs, 1989). The two mains reasons for this difficulty are the requirement to have two domains experts: software and legal, as well as the need to transform the knowledge into a form which can be processed by a machine. An example for the former is the need to have a medical doctor and a programmer for the creation of a medical expert system, while for the second, the need to transform arbitrary knowledge into if-then rules, in order to be able to present it to the user in the form of an expert system.

This need to apply a transformation on the original text in order to obtain machine processable result is an especially complicated challenge from several reasons. It clearly requires more work for creating the knowledge base. More importantly, it is both harder to validate it and to maintain it. Validation is harder as the additional processing made the result more different than the original text and thus makes it harder for the creator of the knowledge base to validate the result. At the same time, maintaining the knowledge base is also harder now, since changes to the legislation require more work in order to identify and apply those changes on the knowledge base. Even systems which are created with a relatively flexible tool, such a programming language, suffer from knowledge transformation.

In their paper, Sergot et al. (1986) have shown how a legal expert system can be created by using the Prolog logical programming language. Others (e.g., Leith, 1986) have pointed to the various problems in such an approach, such as the need to further interpret knowledge in a format supported by the programming language.

Both these challenges have been the focus of research. The need of several domain experts is dealt with mainly via no-code platforms, such as Neota Logic (Mills, 2016) and Visirule (Langley and Spenser, 2007). Nevertheless, they do not solve the second challenge and are therefore hard to validate and maintain.

The second challenge is addressed by providing a richer language in which to capture knowledge. Such languages are usually paired with a reasoning engine, which tries to emulate actual legal reasoning, and normally depend on non-classical logics such as Defeasible Deontic (Nute, 2012) or Higher-order logics (Nipkow et al., 2002).

These systems have the potential to overcome the main disadvantages of expert systems. By utilizing a more sophisticated reasoning engine, they allow for a more compact and faithful representation of the original legislation, while preserving the main advantages of expert systems.

Nevertheless, such systems have enjoyed little commercial success in the legal domain. The main goal of this paper is to investigate the reasons for that and to suggest remedies.

The most obvious shortcoming of such systems is their inability to address the first challenge. In fact, often, the richer the language is, the higher the need is for several domain experts.

This usability issue was identified to be one of the main requirement of expert systems building tools (e.g., Novotná and Libal, 2022). Nevertheless, most such tools fail in achieving this goal (Soavi et al., 2022).

Various attempts have been done in order to make such tools more accessible to legal practitioners via a more user-friendly interface. Logical English for example (Kowalski, 2020), provides an English based controlled natural language interface, which communicates with the underlined logic program. The main advantage of these approaches is the reduced dependency on several domain experts.

Another approach, demonstrated in Robaldo et al. (2020), is by the use of general formats and languages. Being very general, one can more easily utilize a language which is most suited for capturing the semantics of legal texts. One disadvantage of this approach is that these formats do not directly give raise to automated reasoning.

A main additional disadvantage of all the approaches above is the lack of tools and methodologies for asserting the correctness of the logical representations of legal texts. A discussion about the need can be found in Heimtz et al. (2020)^1^.

Among existing validation results, one can find a methodology for building legal ontologies (Mockus and Palmirani, 2017) and more generally for building legal knowledge bases, one for validating formal representations of legal texts (Bartolini et al., 2018).

To summarize, the above approaches underline a key issue in legal reasoning and knowledge representation, which is the tension between the expressiveness of the knowledge and the efficiency of reasoning over it (Benzmüller, 2019). Expressivity refers to the ability of the language to directly capture the nuances of the target language, which in our case is the legal language. Being efficient to reason over refers to the ability to use existing and efficient tools to reason over formulae denoted in this language.

Those approaches which use a programming language as the format for knowledge representation enjoy efficient reasoning but require a non-trivial translation in order to capture the nuances of the law. Moreover, while they are often capable of producing a proof, these proofs are not easily translated into legal arguments. On the other hand, approaches targeting the legal text directly, do not offer a direct reasoning engine.

In the next section, we formalize the above tension using the existing literature and present a running example. We also discuss the lack of objective evaluation methods for the various existing knowledge formalization methods and offer such method.

In Section 3, we propose a new approach to legal formalization, aiming at resolving the tension. We describe how the proposed solution meets the different requirements identified in Section 2 and present an implementation of the new approach.

We conclude with a discussion on a collaboration with an industrial partner to generate a legal knowledge base. We note the advantages of the tool, as well as some disadvantages. We also discuss future work.

## 2. Materials and methods

As discussed in the introduction, legal formalisms suffer from the tension between expressiveness and efficient reasoning. This tension is captured by the following three requirements (Routen and Bench-Capon, 1991):

1. be a faithful representation of what is expressed by the legislation.
2. be computationally adequate, i.e., should permit us to make all relevant derivations by machine.
3. be easy to validate and maintain.

General and extensible formats such as DAPRECO (Robaldo et al., 2020) can clearly achieve point (1) above. But obtaining point (2) seems challenging. For programming languages, the opposite is true, as they are normally computationally adequate. On the other hand, they require a translation between the original text and the programming language and might, therefore, not be a faithful representation.

Both approaches would struggle with point (3). On the one hand, we expect the formalization to be certified by a legal expert. On the other, both programming languages and other formal formats require a programmer or a logician for the encoding. This gives raise to methodologies for validation such as Bartolini et al. (2018).

Maintaining the knowledge base requires the ability to track changes in the law and apply them to the formalization. Any encoding of the law which depends on some manual translation will not be easily maintainable and might introduce errors. A similar problem exists in software engineering, where changes and maintenance of code are a known challenge. Running fully automatic tests is then an essential step in the process (Meyer, 2008). Similarly, legal knowledge bases which depend on arbitrary manual processing steps will be harder to main due to the need to replicate these steps on every change.

The validation of knowledge bases is another hard to achieve requirement. Formal knowledge bases are rarely identical to the original, non-formal, ones. Unlike non-formal ones, formal representations must be non-ambiguous, for example Allen and Engholm (1979). Any syntactical ambiguity in the text must therefore be eliminated in the formalization process, rendering the result different. It is then obvious that the farther the result is from the original text, the harder it is to validate it.

It seems then than none of the current systems can support more than one of the requirements.

Our approach for defining a new system follows the above reasoning. We identify point (3) above as the one currently not adequately supported by existing systems. We then propose a solution which meets the requirements of this point and develop the idea further in order to support the other points.

An important point which is not directly covered by the above three points, but which is often mentioned in discussions with lawyers, is accountability. We consider this point as closely related to validation, as it qualifies what is a good validation process.

DEFINITION 1 (A good validation process). *A good validation process is one which allows the validating lawyer to be accountable for a specific legal formalization*.

In the next subsection, we will introduce our running example, taken from the GDPR.

### 2.1. The data transfer problem

Our running example is based on a collaboration with a German GDPR consultancy^2^, who helped us identify “data transfers” as an interesting problem.

Data transfers are a type of data processing, which have a specific treatment in the General Data Protection Regulation (GDPR). The law governing data transfers is defined in articles GDPR:44–GDPR:49 and also includes European Data Protection Board (EDPB) guidelines, as well as court case decisions, such as Schrems II.

DEFINITION 2 (Original legal text). *When discussing legal texts, the original legal text refers to the text as can be found in regulations, court case decisions, etc*.

For the running example, we will focus on articles GDPR:44 and GDPR:45:1.

EXAMPLE 1. *GDPR:44: “Any transfer of personal data which are undergoing processing or are intended for processing after transfer to a third country or to an international organization shall take place only if, subject to the other provisions of this Regulation, the conditions laid down in this Chapter are complied with by the controller and processor, including for onward transfers of personal data from the third country or an international organization to another third country or to another international organization. All provisions in this Chapter shall be applied in order to ensure that the level of protection of natural persons guaranteed by this Regulation is not undermined.”*

*GDPR:45:1: “A transfer of personal data to a third country or an international organization may take place where the Commission has decided that the third country, a territory or one or more specified sectors within that third country, or the international organization in question ensures an adequate level of protection. Such a transfer shall not require any specific authorization.”*

Basically, the first article declares a prohibition, while the second article offers an exception.

We conclude this section with a suggested methodology for evaluating the approach presented in this paper.

### 2.2. Literature discussion and evaluation plan

In this paper, we argue about the merits of a new methodology and a new tool for the formalization of legal texts. The goal of this methodology though, is to allow jurists without a logic background to create formal knowledge bases and create applications over them. It is therefore essential that the methodology and tool will be compared to other existing approaches in an objective way. While such an experiment for the evaluation of the results in this paper was not yet taken, we have decided to include the description of such an experiment in this section.

This experiment is divided into two elements. We first must be able to select, in an objective way, all relevant tools and approaches. We then suggest an experiment for comparing these tools over established criteria.

This section largely follows the work in Novotná and Libal (2023)^3^.

#### 2.2.1. A methodology for selecting relevant approaches and tools

Our selection methodology is based on searching for research papers describing tools on the Google Scholar search engine.

The search consists of three elements: the resulted artifact, the application domain, and the user interface.

The first problem we encountered is the lack of a fixed vocabulary describing such terms. For example, possible candidates for the resulted artifacts include: program, knowledge base, formalization, specification, controlled natural language, law as code, and others.

In addition, we have found that depending on searching only might be too discriminating and some manual work must be done in order to properly classify all possible publications. We have therefore broken down the selection process into searching and manual filtering.

For the searching phase, we have decided to focus on publications from the following venues, assuming that successful tools will be published there. In addition, we restricted our attention to publications from the period 2018–2022, again under the assumption that even for older tools, in case of some success, follow up papers would be published. Lastly, we have constrained the application domain and intended use of the tools.

[(source:“icail”) OR (source:“jurix”) OR (source:“Artificial Intelligence and Law”)] AND (“legislative” OR “legal” OR “statutes” OR “contracts”) AND (“drafting” OR “interpretation” OR “simplification” OR “comparison” OR “reasoning”) AND after:2018

This resulted in 313 publications, which we then proceeded to filter by hand according to the following categories: tool, methodology, blockchain, machine learning, formal logic, ethics in AI, and survey paper.

In order to be classified as a tool, a paper has to present a new software. Approaches for legal knowledge creation which are based on existing software, such as Prolog (including Zheng et al., 2018; Fungwacharakorn et al., 2021), were classified as methodologies.

After the filtering process, the tools category contained seven papers, two of which were discussing the same tool.

The tools are described in Table 1.

**Table 1:** Selected tools.

**Tool name**	**Interface type**	**Citation**
Logical English	Controlled natural language	Kowalski and Datoo, 2022
Blawx	Drag and drop	Morris, 2021
LegAi	Annotations	Francesconi, 2022
Catala	Programming language	Huttner and Merigoux, 2022
ProLeg	Programming language	Satoh et al., 2021

#### 2.2.2. Criteria for comparing approaches and tools

We design our comparison methodology on the criteria defined in Novotná and Libal (2022) based on the literature review of methodologies for legal formalization^4^. We gathered state-of-the-art approaches and methods of legal formalization and we define the necessary properties for legal formalization method to be practically applicable for legal reasoning. We also view these criteria as suitable formalization features for evaluation. We argue that a good formalization method can be identified according to the extent to which these criteria are met. Furthermore, we consider human evaluation to be the most appropriate evaluation method to assess set criteria. As legal formalizations contain the interpretation of legal texts (in fact, we can even say that a legal formalization IS the interpretation of a legal text) we consider as fundamental to employ legal experts to provide such interpretations. We therefore suggest using a group of legal experts for the evaluation.

We suggest the following criteria for the evaluation of legal formalization methods. A more detailed description of each follows.

1. correctness,
2. transparency,
3. comprehensibility, and
4. multiple interpretations support.

##### 2.2.2.1. Correctness

The *correctness* of the logical representation of a certain legal text and its meaning is indisputably the most important parameter and *conditio sine qua non*. Some research directions use the concept of isomorphism as a mapping between a legal rule and its representation. According to other related research, the *correctness* of a legal formalization is equal to the decision on the interpretation of a legal text as provided by a legal expert (sometimes in cooperation with a logician or a computer scientist).

Defining a single correct interpretation of a legal text is a difficult task even for lawyers and legal experts. For such a legal formalization to be further used for legal reasoning, it is necessary to define a single interpretation for specific circumstances at a particular time. Such interpretations can be based on the legal theory of soft cases and hard cases.

Based on this theory, the vast majority of cases (legal conflicts) are either soft or easy—they can result from the text itself or from the straightforward interpretation of the legal text. Only a small part of legal conflicts require more advanced interpretation methods and the result of the interpretations can be controversial, with several possible reasonable outcomes. We apply this methodology analogically to the interpretation of legal texts for legal formalization. Given this theory, the vast majority of legal rules should be formalized in a non-controversial way and it should be possible to find a broad agreement on a single interpretation. However, there will always be a small part of the legal rules, which will be problematic for formalization because of multiple possible reasonable interpretations. In such cases, we assume that the conflicting elements are abstracted over in order to avoid giving any specific interpretation.

##### 2.2.2.2. Transparency

The *transparent* manner of the translation from a legislation to a logical formulae is necessary for the assessment of all of the other parameters. In cases where mapping the logical relationships among legal terms in the original legal text and encoding them in logical formulae usually requires two experts—a logician (or a computer scientist) and a legal expert—such transparency helps them understand each other process. More generally, such transparency allows others, as well as the author itself, to trust the formalization process and the resulted knowledge bases.

##### 2.2.2.3. Comprehensibility

The *comprehensibility* of a legal formalization is closely related to its *correctness* and its *transparency*. Although these three terms are separated, their evaluation will often overlap in practice. The *comprehensibility* of a legal formalization lies in a general understanding of the method and its result, i.e logical formulae. Where the *transparency* parameters should evaluate the relationship between the original text and its logical representation, the *comprehensibility* parameter should evaluate the complexity of the logical representation as an output. The *comprehensibility* of such an output is necessary for the evaluation of a logical formalization as well as for the broader use of the evaluated methodology.

Simply put, a logical formalization which is difficult to read, analyze or understand is not very suitable to be used in practice by lawyers or laymen. In this regard, this parameter is closely related to the friendliness of a user interface and the presentation of the formalization. We believe that a more comprehensible output of the legal formalization is a crucial step toward a wider use of the methods and large-scale evaluations and therefore, toward more significant results.

##### 2.2.2.4. Multiple interpretation support

As was described above, the *support of multiple interpretations* for a single legal text is necessary for several reasons. There is an extensive literature body related to the ambiguity and vagueness of legal texts and very often the legal discourse itself does not agree on a single correct interpretation. Additionally, there are well-described legal and extralegal circumstances causing the ever-changing characteristics of the law.

It is very common, that a generally accepted interpretation of a certain legal rule changes in the context of related higher court decisions even in continental legal systems. Furthermore, there are social changes and novelizations of legislation which change the interpretation every now and then. Therefore, systems which are rigidly dependent on one interpretation of a legal text, which is moreover highly laborious, will always be limited for use and are very probably highly maintenance intensive.

This situation favors systems and methods which are *dynamic*. This means that the formalization can be easily changed or it can *support several interpretations* of single legal text at once. We suggest evaluating this as a further parameter of legal formalization methods.

#### 2.2.3. Designing an experiment for comparing approaches and tools

According to the description of the criteria we defined in the previous section, we propose here a methodology for an objective evaluation experiment based on the use of a legal expert group. In this methodology, we focus mainly on the practical side of legal formalization. That means we prioritize an interaction with legal experts and therefore, we employ a user-oriented approach in the evaluation experiment design. We believe that for a legal formalization method and its associated tools to be widely used and accepted, such a method must be intuitive and easy to use.

Such an approach brings several advantages—it is less time-consuming and costly, it can be used repeatedly with different legal experts or as a collaboration, the formalization is not rigid but can be easily changed along with the development of the meaning of the legislation and it can be applied to different legislative texts (i.e., it is not tied to only one document), to name but a few.

Our approach is based on the evaluation of the user experience when formalizing a specific legal text. For the experiment itself, a careful choice of the relevant legal source and reasoning should be done. We plan on choosing a short section of a regulatory text (article, paragraph) containing different types of legal modalities in the context of other legal institutes. In addition, we will select a part of a court decision which depends on the regulatory text. Such an exemplary regulatory and case law sources should be presented to a group of legal experts for formalization and evaluation.

Manual-oriented evaluation of methods and tools consists of instructing a group of legal experts to use the evaluated tools in order to translate the example text into logical formulas. To make the results of such an experiment as meaningful as possible, it is necessary to have as large and ideally diverse group of experts as possible. That is, a group comprising both experts in the particular legal sector related to the regulatory text in the example and experts from other legal sectors.

The legal experts must be properly instructed in the use of each tool evaluated. To this end, each tool or method evaluated should be accompanied by authentic documentation and a methodology for its use. At the same time, the instructions on the use of the different methods should be pre-drafted by the authors of the study so that there are no significant differences between them that would then affect the evaluation of the different methods.

The group of legal experts is then asked to formalize the submitted text using the evaluated tools. The evaluation of this formalization would then be carried out using the criteria defined previously—evaluation of the correctness of the formalization, evaluation of the comprehensibility of the tool, evaluation of the transparency of the method and evaluation of whether the tool supports the multiple interpretations.

We intend the evaluation itself to consist of a scalable questionnaire containing various questions over these criteria. The correctness of the formalization is assessed by the consensus of legal experts on whether the formalized text corresponds to the original meaning of the text. The comprehensibility of the tool would be evaluated following the user experience of working with the tool and the comprehensibility of the related documentation. The transparency of the method would be evaluated by assessing the relationship between the original text and its representation and whether this relationship is clear to the evaluators. Support for multiple interpretations would then ideally be evaluated as a characteristic of the tool and the ease of changing the logical representation according to different interpretations.

## 3. Results

In this section, we describe a possible solution to the tension described in the previous section and present an implementation. In the next subsection, we will define a formalization language which attempts to bridge between the two main requirements: expressivity and computational adequacy. We then describe the different properties of this approach and conclude with an implementation, freely available to users.

### 3.1. Legal linguistic templates (LLTs)

This section is based on the meta-level annotations introduced in Dastani et al. (2020) and extends the joint work in Abidi and Libal (2022).

If we consider the formalization processes as a function from legal texts to logical formulae, the validation requirement states that this function must be isomorphic. Given a formula, we should be able to generate the original text.

In order to achieve that, we need a function which is as lossless as possible. Any translation function which loses information in the process cannot be inverted. Therefore, it seems that the only possible solution to that is to use a logic which is as expressive as the original text. This also implies point (1). If the logic is as expressive as the original, then regenerating the original text closely amounts to just pretty printing formulae. Using a logic of lower expressivity would cause any translation into the original text to result in some level of information loss.

In order to define such a logic, we have discussed with data protection lawyers the way they would interpret the original legal text.

DEFINITION 3 (Legal interpretation). *A legal interpretation of a legal text is defined as the interpretation of a legal expert with regard to a specific context problem. It should be noted that interpretations are often subjective and context dependant*.

Our lawyers gave the following interpretation to the articles in the running example.

EXAMPLE 2. *Articles GDPR:44 and GDPR:45:1 are interpreted as follows:*

- *GDPR:44—The data processor is generally prohibited to transfer personal data to a third country or an international organization*.
- *The data transfer to a third country or an international organization, mentioned in GDPR:44, is allowed if the European Commission has made an adequacy decision with regard to this country or organization*.

It is important here to comment further on the meaning of a legal interpretation. The definition above sets it as subjective and context dependant and in the remaining of the section we will describe how it can be achieved. We will not discuss further though questions such as the ability to denote ambiguity in the interpretation. We would like therefore, to briefly discuss how this can be obtained using a logical language in general.

In order to preserve the ambiguity of a legal interpretation, i.e., allowing it to denote more than one meaning, one can use the notion of abstraction. Edelman (1992) discusses the concept of ambiguity in law and gives the following example, based on title VII of the American Civil Rights Act.

SEC. 703. (a) It shall be an unlawful employment practice for an employer: (1) to fail or refuse to hire or to discharge any individual, or otherwise to discriminate against any individual with respect to his compensation, terms, conditions, or privileges of employment, because of such individual's race, color, religion, sex, or national origin; or (2) to limit, segregate, or classify his employees in any way which would deprive or tend to deprive any individual of employment opportunities or otherwise adversely affect his status as an employee, because of such individual's race, color, religion, sex, or national origin.

Edelman then further states two possible interpretations: (1) A procedural interpretation referring to the treatment; or (2) a substantive interpretation focusing on the outcome. An attempt to clearly formalize the concept of discrimination in either of the above ways might exclude the other.

Logic equips us with two tools to deal with such problems. One can first denote the two possible interpretations by the use of a disjunction—discrimination can be either dealt with procedurally or substantially. This approach might be limited given the open texture of the law (Schauer, 2013), which allows for interpretations to be made later and under different contexts.

Another way for dealing with ambiguity, which is consistent with the open nature of the law, is by the use of abstraction. In the above example, one just refrains from making a choice between the two interpretations and instead might set specific conditions for each one of them, those leaving the concept abstract in all other cases. This is another example of the tension between representation and reasoning discussed all along the paper. As a simple example of this concept in action, please refer to Section 3.5.

Lastly, we would like to briefly comment on the ability to formalize legal sources in the face of varying, sometimes contradicting, legislations. Various solutions have been offered to overcome this problem when formalizing legal texts. Among them, Alchourrón and Makinson (1981) have proposed partially ordering the precedence of different legal sources, a concept which can be captured by relatively weak logics.

In order to formalize legal interpretations, we therefore need a language expressive enough to capture legal structures, while at the same time, be computationally adequate. A common solution to this problem is by the use of Domain Specific Languages (Mernik et al., 2005).

At the same time, this language needs to capture subjective interpretations and should therefore be flexible enough to meet the needs of different users. We have therefore opted for the use of a templates language which can be extended with customized templates, according to the needs of users.

DEFINITION 4. [Legal Linguistic Templates (LLTs)] *Legal Linguistic Templates consist of a name and one or more parameters*.

An LLT which forms the basic of all LLT languages is the Atom.

DEFINITION 5 (The Atom LLT). *Atoms are LLTs containing* *n*+1 *parameters. The first is the name of the Atom and the remaining* *n* *parameters are its arguments. An Atom with name* *f* *and arguments* *a*_1_, …, *a*_*n*_ *is written as the first-order (see for example Barwise*, *1977**) term* *f*(*a*_1_, …, *a*_*n*_) *and can be defined inductively by:*

- A symbol *f* is an Atom
- If *a*_1_, …, *a*_*n*_ are atoms with names *b*_1_, …, *b*_*n*_, respectively and *c*_1_, …, *c*_*m*_, *f* are symbol, then *f*(*b*_1_, …, *b*_*n*_, *c*_1_, …, *c*_*m*_) is an Atom.

EXAMPLE 3. *The following Atoms relate to the running example:*

- label(name)
- dataProcessor(name)
- dataSubject(name)
- personalData(dataSubject,name)
- thirdCountryOrInternationalOrganization (name)
- dataTransfer(dataProcessor,personalData, thirdCountryOrInternationalOrganization)
- adequacyDecision
- (thirdCountryOrInternationalOrganization)

Our representation of Atoms as first-order terms is restricted to shallow terms only, i.e., terms which do not contain nested terms as arguments. In addition, by omitting quantifiers from the language, we make an explicit decision as to how the arguments (which are universally quantified variables) are bound within the knowledge base. Our decision is to bind them on the level of each sentence, in a similar way to the implicit quantification in logic programming languages such as Prolog.

EXAMPLE 4. *The LLTs language which can express the legal interpretation in the running example can be inductively defined by:*

- An Atom is an LLT
- If A is an Atom and B is an LLT, then LabeledStatement(A,B) is an LLT
- If A is an LLT, then GeneralProhibition(A) is an LLT
- If A_1_, …, A_*n*_ are atoms and P is an LLT, then Condition(A_1_, …, A_*n*_,P) is an LLT
- If A and B are Atoms, then Exception(A,B) is an LLT

We can now define formal interpretations of legal ones and use the above language to represent the interpretation of the running example.

DEFINITION 6 (Formal interpretations). *Given a legal interpretation* *L* *containing* *n* *statements, a formal interpretation for* *L* *is a set of* *n* *statements over an LLT language and a set of atoms*.

EXAMPLE 5. *Given the LLT language, atoms and legal interpretation of the running example, a formal interpretation is the following:*


    LabeledStatement(
      label(gdpr:44),
      Condition(
        dataProcessor(DP),
        dataSubject(DS),
        personalData(PD,DS),
        thirdCountryOrInternationalOrganization
        (TC),
        GeneralProhibition(dataTransfer(DP,
        PD,TC))))
    Exception(
      label(gdpr:44),
      adequacyDecision(TC))
    


Before we move forward to the computational adequacy of the LLTs language, we would like to mention that within the terminology of programming languages (Pierce, 2002), a formal interpretation can be reduced into an abstract syntax tree over a first-order term language.

### 3.2. The computational adequacy of LLTs

By choosing an LLTs language which is expressive enough to describe a “good” formal interpretation of a legal text, we seem to go further away from computational adequacy. In this subsection, we show that this is not necessarily the case.

We achieve that by following the “shallow semantical embeddings” defined by Benzmüller (2019). The basic idea of this approach is to provide a lean and elegant equational theory which interprets the syntactical constituents of logic L (in our case a specific LLTs language) as terms of another logic M.

If the logic M that we choose is computationally adequate, then we effectively obtain the computational adequacy of L. This is achieved in practice by applying the shallow semantical embedding, and then applying software for the computationally adequate result.

The logic chosen by Benzmüller is Higher-order Logic (Church, 1940) and the various software which can be used to reason over the embeddings include Isabelle/HOL (Nipkow et al., 2002) and Leo-III (Steen and Benzmüller, 2018).

The LLTs languages we have used so far have required an embedding to a much simpler and computationally adequate logic. In this section, we describe a simplified logic adequate for the running example.

We will also generalize the notion of a “shallow semantical embedding.”

DEFINITION 7 (Embedding functions). *An embedding function* ϕ *of a logic L into a logic M, is a total function from L to M*.

DEFINITION 8 (Meta-logic). *A meta-logic is a computationally adequate logic, into which an embedding function from another logic exists*.

EXAMPLE 6. *First-order horn clauses with negation interpreted as negation-as-failure (NAF) (Clark*, *1978**) form a meta logic as it can be executed in Prolog (Colmerauer*, *1990**)*.

We can now give an embedding function between the LLTs language in the running example into first-order horn clauses with NAF.

EXAMPLE 7. *The following is the inductive definition of an embedding function from an ordered set of LLTs, which were defined in the running example, into a set of first-order logic with NAF formulae, where NAF is denoted by* not.

- ϕ(∅)⇒∅
- ϕ({*Atom*})⇒{*Atom*}
- ϕ(*S*∪{Condition(*a*_1_, …, *a*_*n*_, *p*)})⇒{ϕ(*a*_1_), …, ϕ(*a*_*n*_)⇒ϕ(*p*)}∪ϕ(*S*)
- ϕ(*S*∪{GeneralProhibition(B)})⇒prohibited(ϕ({*B*}))∪ϕ(*S*)
- ϕ(*S*∪{Exception(A, B), LabeledStatement(A, C)})
- ⇒{notϕ({*B*})⇒ϕ({*C*})}∪ϕ(*S*)

*Note that the resulted set denotes a set of first-order formulae, which might not be a horn clause. A standard normalization can be used in order to obtain a logic program where arguments of atoms are universally quantified on the level of individual expressions. The function* prohibited*is uninterpreted*.

Having the embedding, we can now use Prolog in order to compute various properties. To check for violations of the prohibition, for example, one would need to add Prolog facts such as dataTransfer(a,b,c) and check if prohibited(_) can be derived.

We finish this section with the Prolog code of the formal interpretation of the running example.

EXAMPLE 8. *The Prolog code for the formal interpretation of the running example is the following*.


    prohibited(dataTransfer(DP,PD,TC)) :-
      dataProcessor(DP),
      dataSubject(DS),
      personalData(PD,DS),
      thirdCountryOrInternationalOrganization
      (TC),
      not adequacyDecision(TC).
    


### 3.3. Validation of LLT formalizations

The last requirement of a legal formalization language is to have a good validation process, which can ensure that legal experts be held accountable.

The approach taken in order to meet this requirement follows a very basic idea in programming languages, that of a pretty printer (Hughes, 1995). Pretty printing takes a formal structure, such as an LLT formula, and presents it in a user friendly form.

While the concept is basic, it turns out to be very powerful in the legal domain. By printing an LLT formula as a legal statement in English, we effectively translate it back into the original legislation language. This feature allows a legal expert to compare the two versions of the legislation—the original and the pretty printed one—and to make a decision regarding its validity.

The idea of reverse translations is not new. Translators are using a reverse translation in order to evaluate the quality of machine translation. By completing the translation circle back to the original language, the task of validating the translation becomes easier. Similarly, reverse translations are used in order to evaluate machine learning algorithms (Kornilov et al., 2021).

The evaluation of legal reverse translations can be made even when the legal expert does not have any knowledge in logic and has not participated in the generation of the LLT formulae.

DEFINITION 9 (Reverse translations). *Given an LLT language and a legislation in a specific language, a reverse translation for this language is a function from LLT formulae into the specific language*.

As an example, consider the following reverse translation.

EXAMPLE 9. *Given a translation* ξ *of Atoms, the following recursive function* ψ *is a reverse translation for the running example (see Def*. *4)*.

- ψ(Atom) = ξ(Atom)
- ψ(LabeledStatement(A, B)) = ψ(A)]ψ(B)
- ψ(GeneralProhibition(A)) = It is generally prohibited thatψ(A)
- ψ(Condition(A_1_, …, A_*n*_, P)) = Given thatψ(A_1_), …, ψ(A_*n*_)thenψ(P)
- ψ(Exception(A, B))
  = Statementψ(A)does not hold in case of
  ψ(B)

To complete the example, we define ξ.

EXAMPLE 10. *The following function defines a translation of the atoms in the running example (where “thirdCountryOrInternationalOrganization” is abbreviated as “cio”)*.

- ξ(label(name)) = name
- ξ(dataProcessor(name)) = Data Processor (name)
- ξ(dataSubject(name)) = Data Subject (name)
- ξ(personalData(dataSubject, name))) = Personal Data (dataSubject, name)
- ξ(cio(name))= Third Country or an International Organization
(name)
- ξ(dataTransfer(dataProcessor, personalData, cio))=
Data Transfer (dataProcessor, personalData, cio)
- ξ(adequacyDecision(cio)) = Adequacy Decision by the European Commission (cio)

*The names in parentheses define the parameters of the vocabulary*.

Finally, applying the reverse translation to the formal interpretation of the running example results in the following two sentences.

EXAMPLE 11. *The resulted reverse translation of the formal interpretation of the running example is (parameters omitted for brevity):*


    gdpr:44] Given that
        Data Processor,
        Data Subject,
        Personal Data,
        Third Country or International
        Organization
      then It is generally prohibited that
        Data transfer
    Statement gdpr:44 does not hold in case of
      Adequacy Decision by the European
       Commission
    


The reverse translation above, which is done for illustration purposes, still clearly lacks proper readability. Nevertheless, generating a linguistically correct version out of the above sentences is not a difficult task for current machine learning algorithms. It should be noted though that the statistical errors associated with these algorithms are limited to readability only and do not effect the legal correctness.

For completeness, we present one such possible translation, similar to the one automatically generated by the tool presented in the next section.

EXAMPLE 12. *A human readable reverse translation can be created as follows*.


    gdpr:44] The Data Transfer of Personal Data
      to a Third Country or International
      Organization
      is Generally Prohibited.
    The Transfer of Personal Data to a
    Third Country
      or International Organization is
      allowed if there
      is an Adequacy Decision by the
      European Commission.
    


### 3.4. The LegAi editor—An LLT implementation

In this section, we present an implementation of an editor for creating LLTs, as well as using them to annotate legal texts and to validate the quality of the annotation.

In Section 4, we discuss the experiences of a lawyer who has used the system. Since this experience does not constitute an objective evaluation, we have described a proposed evaluation experiment in Section 2.2.1.

The goal of the LegAi annotation editor is to support annotating legal texts with LLTs. At the same time, the editor supports a validation mechanism for these annotations, which allow legal experts to verify the correctness of the annotations.

The editor can be found online, at https://legai.uni.lu, and requires a registration. After registration, the editor displays a message that an email to activate the account must be sent to the code maintainer, who associates the new user to a specific account with specific rights.

The basic entities supported by the editor are **Companies**. Companies contain a list of **Legislations** and have specific sets of LLTs and vocabulary. A company can contain **administrator** and **editor** users. In the following a brief introduction to the functionalities available to each of the users is described.

#### 3.4.1. Editor users

**Figure 1:**
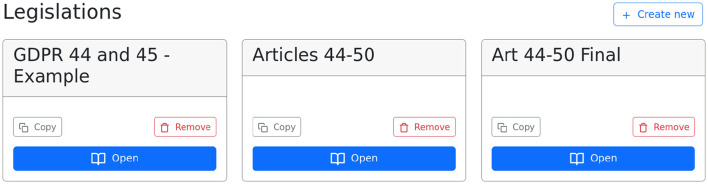
Dashboard for creating and editing legislations.

An editor has access to the LegAi annotation editor and can edit any of the company's legislations. A legislation corresponds to one textual legal document and can be exported into JSON using LegAi's API access. Figure 1 shows the initial editor dashboard. The editor can choose to open, remove, or create a new legislation.

**Figure 2:**
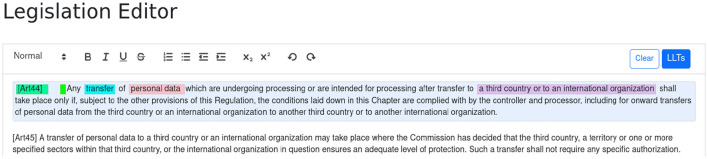
The annotation editor.

Once a legislation is created or opened, the user is presented with a textual editor where the original legal text can be pasted. The user then selects a sentence and clicks the LLTs button in order to start the annotation wizard. Figure 2 shows the editor after the two articles from the running example have been pasted into and article 44 already annotated.

**Figure 3:**
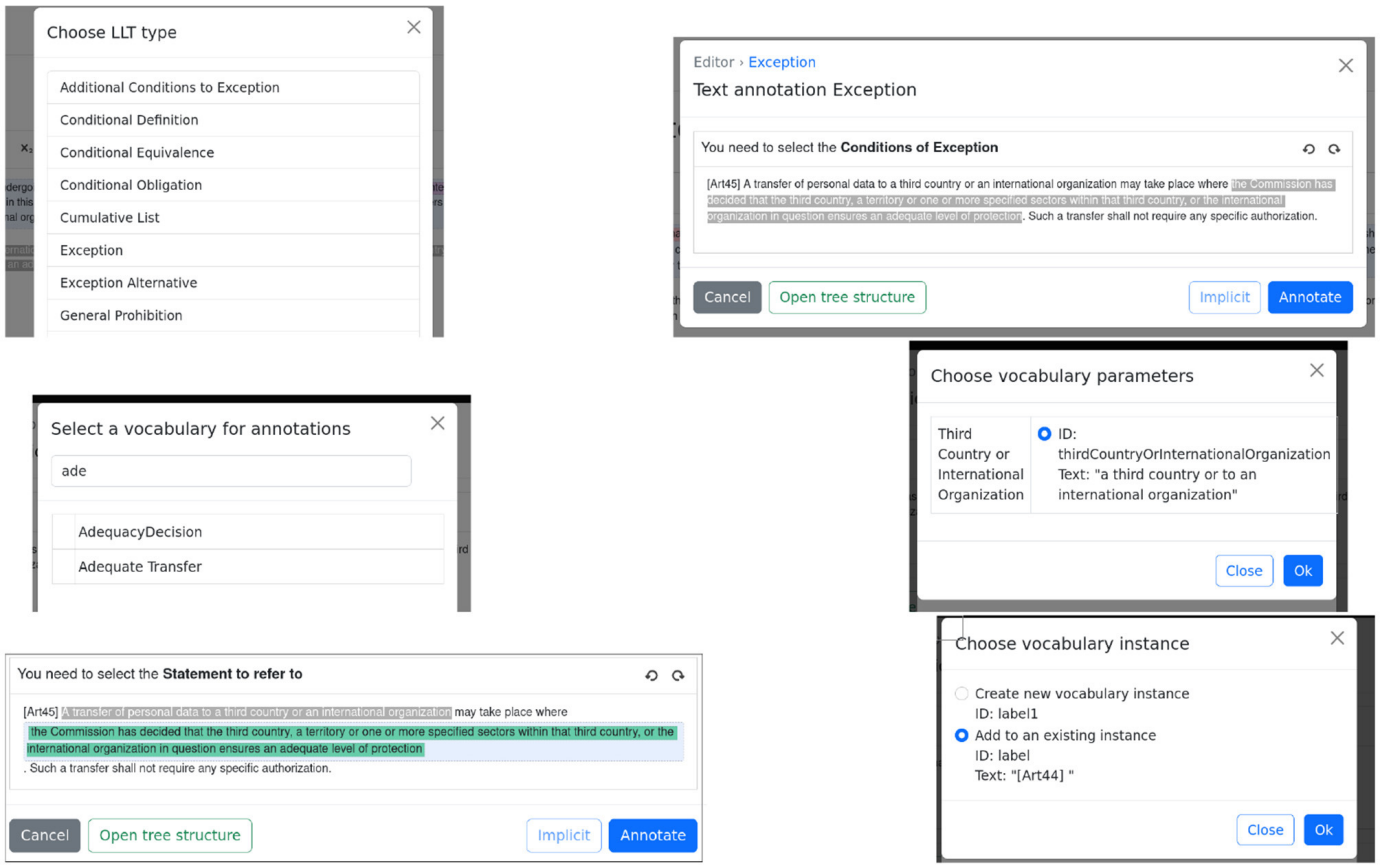
The annotation process of article 45.

We will now proceed with the step-by-step process for annotating article 45, shown in Figure 3. The process progresses from top to bottom and from left to right. The wizard guides the user in the annotation process and shows the possible/required LLTs that can/must be nested. The wizard ensures that annotations are always syntactically correct.

Once the user has selected the text to annotate and clicked the LLTs button, a list with all supported LLTs is displayed. The user then selects the **Exception** LLT and is asked to select the **Conditions of Exception**.

Once the text corresponding to the conditions is chosen as well as further LLTs corresponding to whether the conditions are cumulative or non-cumulative (not shown in the figure), the user is asked to choose which vocabulary corresponds to the condition.

The user chooses **AdequacyDecision**. As adequacy decisions are parameterized by an instance of a third country or international organization, the user is further asked to choose from these instances already used in the legislation. In this case, only one option is available, which corresponds to the same instance used in article 44.

In the last row, the user is asked to specify the **Statement to refer to** and selects the whole text describing the prohibition from article 44, which is repeated in the body of article 45. The user is then asked to choose the label of the already used statement and chooses the one corresponding to [Art44].

**Figure 4:**
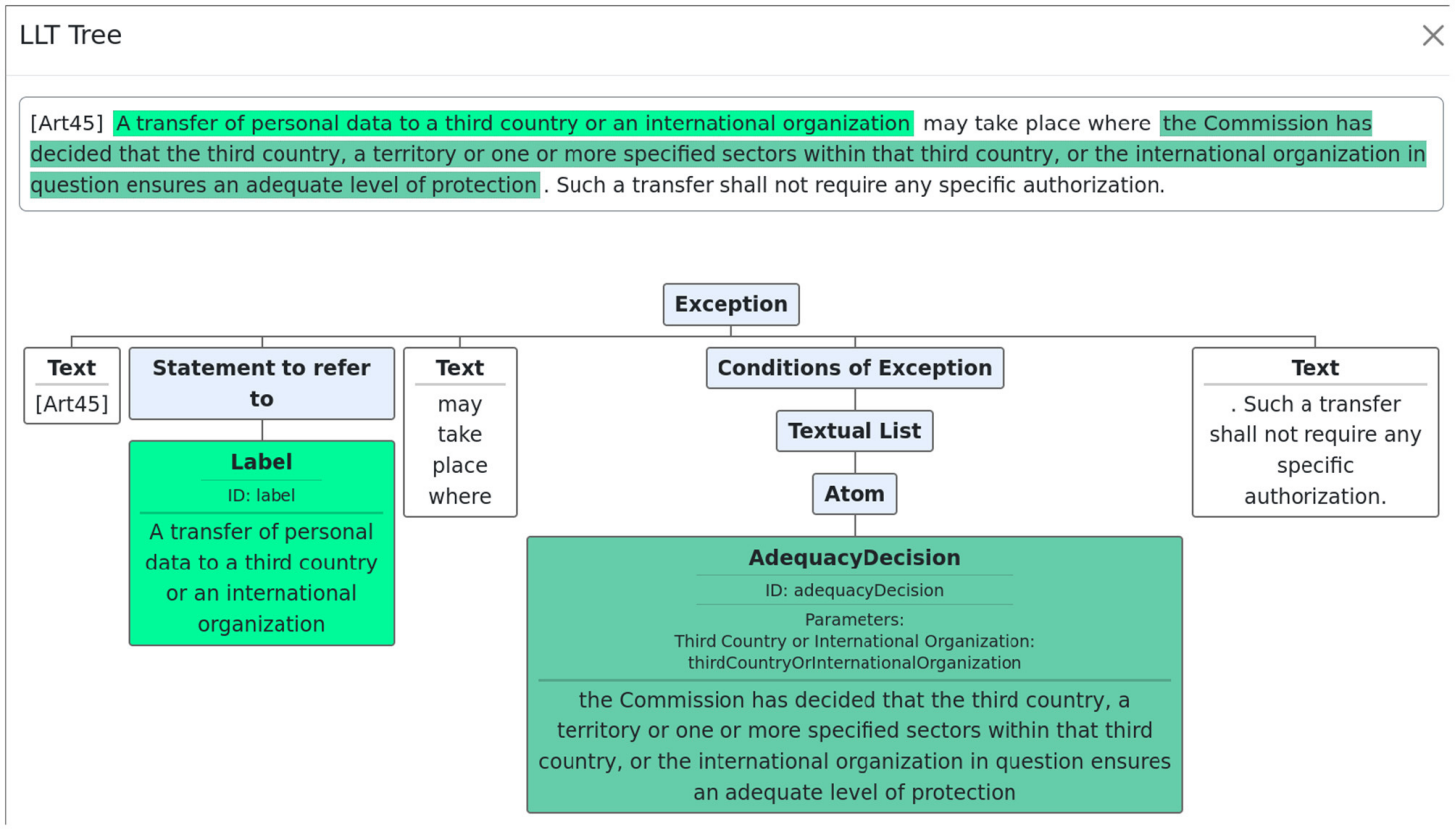
Tree structure of article 45.

Once a top-level LLT is created, the user can click on the annotation and see a tree structure corresponding to the formal version of the text. Figure 4 shows the tree associated with article 45.

**Figure 5:**
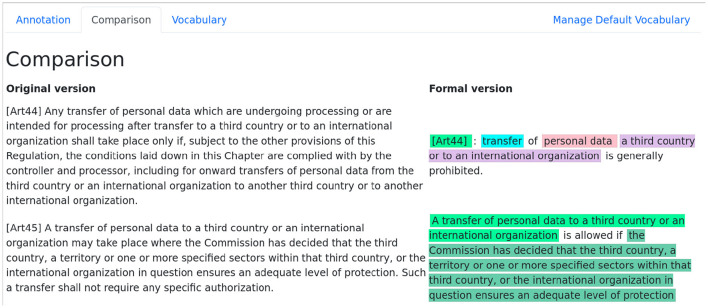
Reverse translation for articles 44 and 45.

Lastly, the user can click the comparison tab, shown in Figure 5, in order to see the reverse translation of the text, next to the original one. A list of all vocabulary and their associated legal texts appear on a separate tab.

#### 3.4.2. Administrator users

An administrator is capable of adding editor users, as well as of creating and maintaining the LLT and vocabulary lists. Adding editors is straightforward and we will focus in this section on the mechanism for creating and maintaining LLTs and vocabulary.

**Figure 6:**
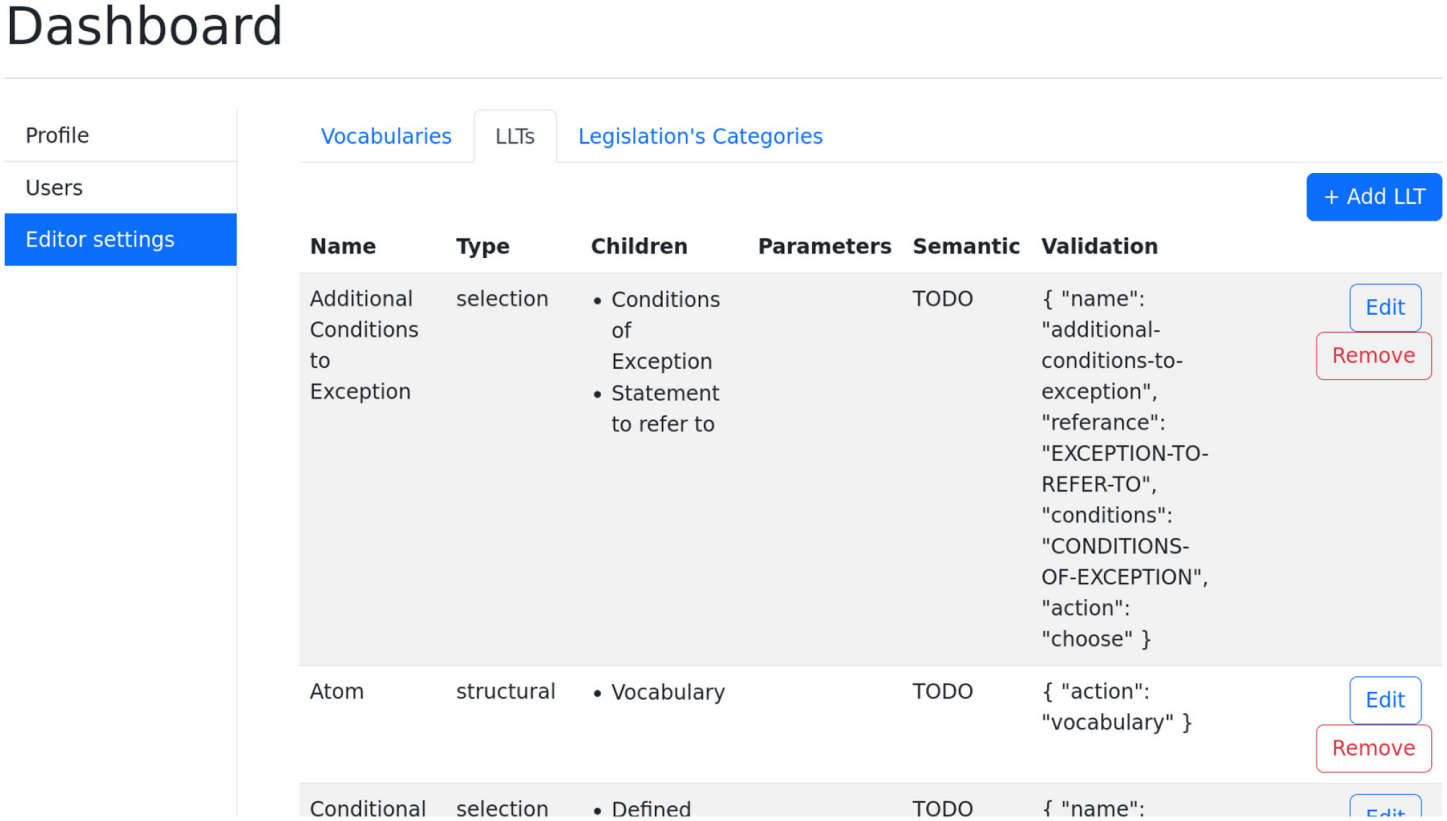
Dashboard for creating and maintaining LLTs.

Figure 6 shows the main LLT dashboard. It displays the available LLTs in a table format. The table also contains information regarding the type and children of each LLT, as well as a JSON template which can be used in order to format the result. The purpose of this dashboard is to allow administrators to create arbitrary LLT structures.

The two different types which are supported by the tool are **selection** and **structural**. When considering the wizard in the previous section, one notices that between concrete LLTs such as Exceptions, Cumulative Lists and Atoms, there are informational steps such as “Specify the Statement to Refer To” and “Select the Conditions of Exception.” The former are of type selection LLTs while the later are of type structural LLTs. This distinction allows for the creation of complex wizards without the need of programming.

Children refer to the LLTs which follow the one currently processed. A selection LLT is normally followed by structural LLTs. Having more than a single structural LLT means that the wizard will ask the user to annotate each of them in parallel. Having more than a single selection LLT means that the wizard will show a list of all available selection LLTs once the text was selected. Together, they allow for the creation of arbitrary LLT structures.

LLTs can have additional parameters, which are specific vocabulary needed for the instantiation of a specific LLT. For example, a temporal condition LLT might require a time vocabulary on instantiation. In addition, restrictions can be placed on the number of children. For example, requiring at least two selection children for a cumulative list.

Lastly, the **Validation** column contains a JSON template for the generation of the final JSON object in the knowledge base. These templates have various types which allow a more concise and readable version of the knowledge to be automatically produced.

In addition to LLTs, administrators also control the list of vocabulary available to editors.

**Figure 7:**
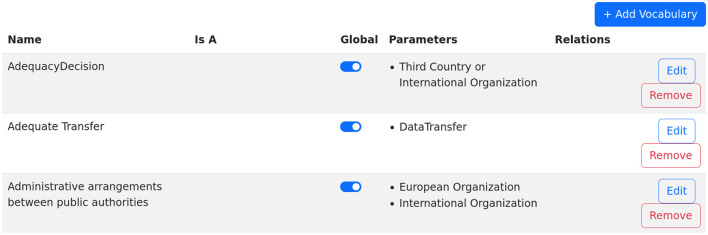
Dashboard for creating and maintaining vocabulary.

Figure 7 shows the dashboard for creating and managing vocabulary. The table contains information about the name of the vocabulary, as well as about their relationships with other vocabulary.

There are two types of such relationships, **is A** is used to express that a vocabulary is a refinement of another vocabulary. **Parameters** are used in order to denote dependency on other vocabulary. For example, an Adequacy Decision is parameterized by a third country or organization, while a Data Transfer is a refinement of a Data Processing (not shown in the figure).

### 3.5. Use case

We conclude this section with a description of a use case. While the use case is not, in any form, a comparative experiment, it might help to highlight the advantages and disadvantages of the method described in the paper.

As part of our collaboration with a GDPR consultancy and the identification of personal data transfer as our compliance problem, we then sat with a data protection lawyer in order to formalize the relevant legal sources. The process we have undertaken is described next.

#### 3.5.1. Identification of the relevant legal sources

The first step in the process was to analyze the relevant legal sources needed for deciding arbitrary compliance problems. The lawyer has identified articles 44–50 of the GDPR, as well as European Data Protection Board guidelines (EDPB)^5^ and Court of Justice of the European Union (CJEU) rulings^6^. Within the formalization process that followed, some details, especially from the guidelines, were deemed too specific and were not included in the formalization. This choice between abstraction and specification displays a fundamental element of legal formalization, namely, the inability to have a complete formalization of some laws. While completeness might not be achieved, soundness was never compromised and was preserved by using abstraction. As an example of different levels of abstraction, consider the term Purpose of limitation mentioned in the GDPR and its specific definition within the above mentioned EDPB guidelines.

#### 3.5.2. Vocabulary extraction

The second step was to identify vocabulary which is not yet defined in the LegAi editor. While many concepts such as Data subject, Data processor, and Data processing were already defined, concepts specific to data transfer were not. In addition to identifying further concepts such as Data transfer and Adequacy decision, one also needed to identify properties such as is-a relations and parameters. As an example of the first, Data transfer was identified as being a type of Data processing. A parameter of Adequacy decision is Third country or international organization.

#### 3.5.3. LLTs extension

This step involves the identification and definition of new Legel Linguistic Templates, which are needed for the correct formalization of the source legal texts. Since the current use case is among the first conducted using the tool, quite a few new LLTs were defined. We believe this would no longer be the case once substantial work was done using the tool. As an example of a new LLT which was defined, we have General Prohibition. The semantics of this LLT are allowing later exceptions to cancel the prohibition stated in the annotation. Its sole child structural LLT is the “Statement” LLT.

It is important to note here that the lawyer could choose various ways of capturing the meaning of the legal text. For example^7^, instead of stating article 44 as a prohibition and 45 as an exception, the lawyer could state article 44 as a general principle and 45 as enshrining a safe harbor.

The point to make here is that extending the LLTs library is not only a choice of what was previously available but also a choice of the preferences of the user. Saying that, LLTs are formal concepts with a precise an unambiguous meaning and it might be that different LLTs will have identical semantics when used for automated legal reasoning.

#### 3.5.4. Iterative annotation and validation

The last step involved the annotation of the text by the use of the vocabulary and LLTs. This step was conducted iteratively, while after every iteration of an annotation of one sentence, the lawyer has checked the reverse translation of the formalization and has updated the annotation if needed. This process has required training in the use of the tool and was sometimes disrupted by the need to define new vocabulary and LLTs.

## 4. Discussion

Legal knowledge representation is essential for various applications, such as semantic search and reasoning. Nevertheless, there is no agreement on the format, logic, and validation method for obtaining the knowledge bases, which are essential for those application.

In this paper, we visit one element of this disagreement, the tension between expressivity and computational adequacy. We offer a solution in the form of an expressive LLTs language and argue that, in some practical contexts, is computationally adequate. In addition, we have shown how this language promotes a validation method and does not require the involvement of logicians or programmers.

In order to test this approach, we have implemented it in the form of a freely available web application and have experimented with an industrial partner for several months. We also envision a more objective experiment, details of which were described in Section 2.2.1.

Our initial findings from the collaboration with the industrial partner seem promising. We have also identified some issues, which need to be overcome for a widespread adaption of such approach among jurists.

In our collaboration with the partner, we have identified checking “personal data transfers” for compliance as our use case. Our first step was to analyze, together with the lawyer, the relevant articles, guidelines and court case decisions.

The lawyer then proceeded to identify the different legal linguistic templates used by the different statements. Besides the “general prohibition” and “exception” which are shown in the running example, we added also many other templates, such as conditional and non-conditional definitions. Definitions are used to make an abstract notion, such as “appropriate safeguards”, more concise.

At the same time, the lawyer has compiled a list of relevant vocabulary and the relationships between them. At this point, we have compared the vocabulary list to the official one from W3C^8^ and standardized it. The lawyer then started with the annotation process, while continuously checking the result via the reverse translation interface.

The most time consuming part was the analysis of the text in order to identify the relevant legal linguistic templates, as well as the extraction of vocabulary. We hope that the variety of legal linguistic templates is rather limited and therefore, that the process of identifying them will no longer be required when some initial knowledge bases have been established. Similarly, the process of extracting vocabulary is needed only when considering a new legal domain.

The main drawback of the methods and tools described in this paper is the need to use universally quantified variables. One difficulty is that the concept of parameterized vocabulary was not so easy for a non-logician to work with. Another difficulty is that the tool we have created struggled with the best way to display and use them.

Our choice at the end was to try to automate the selection of these parameters as much as possible (for example, when only one option is available) and to display this information on the reverse translation tool only as an additional text accessible by hovering with the mouse over the relevant vocabulary instance.

Still, whenever more than one option was available, the lawyer has contacted our team for confirmation. We consider an interface for using and displaying variables and parameters as a main future challenge which we need to overcome before a wider usage of the tool by jurists will be possible.

In the remaining of this section, we will discuss some other future plans and challenges.

The first future work is conducting the experiment, which was described in Section 2.2.1. Currently, there are not enough detailed experiments papers, where prospective users describe the strengths and weaknesses of various systems. In addition, the fact that these systems are normally created by people with a logic background may be the reason why knowledge presentation and legal reasoning are not as successful in the legal domain as in some others.

Another problem which we have identified and plan on investigating in the coming years relates to possible applications of the knowledge bases. This topic was not discussed in the paper, as one of our main claims is the ability to separate the knowledge base from possible applications. Nevertheless, if the goal is to make justice accessible to non-jurists, then the first challenge is the fact that the legal interpretations captured by using tools and approaches such as the ones presented in this paper contain many uninterpreted concepts. Take for example the concept of “appropriate safeguards,” which requires balancing different concepts and is probably not interpretable by non-jurists. We therefore plan on investigating, not only how to capture high level legal interpretations, but also ones which can be easily understood by laymen.

Lastly, we recognize the fact that specific use cases and collaborations with prospective users were essential to the development of the current approach. We plan on continuing the collaboration with stakeholders both in the industry and the academy.

## Data availability statement

The original contributions presented in the study are included in the article/supplementary material, further inquiries can be directed to the corresponding authors.

## Author contributions

The author confirms being the sole contributor of this work and has approved it for publication.

## Footnotes

^1^See discussion in Section 3.2 in Heimtz et al. (2020)

^2^We want to thank Silvan Jongerius, the managing partner of the firm, as well as Ali El Hajj Sleiman, a data protection lawyer and intern at the firm.

^3^This is an ongoing work and we plan a followup once the experiment is concluded.

^4^This is an excerpt from the authors' publication cited below.

^5^Such as Guidelines 2/2020 on articles 46:2:a and 46:3:b of Regulation 2016/679.

^6^Mainly Schrems I and Schrems II with relation to the European Commission adequacy decision with regard to the United States of America.

^7^We thank the anonymous reviewer for suggesting this example.

^8^ https://w3c.github.io/cg-reports/dpvcg/CG-FINAL-dpv-20221205

## References

[B1] Abidi A. Libal T. (2022). “A validation process for a legal formalization method,” in Workshop on Methodologies for Translating Legal Norms into Formal Representations (Saarbrücken).

[B2] Alchourrón C. E. Makinson D. (1981). “Hierarchies of regulations and their logic,” in New Studies in Deontic Logic: Norms, Actions, and the Foundations of Ethics, 125–148. 10.1007/978-94-009-8484-4_5

[B3] Aletras N. Tsarapatsanis D. Preoţiuc-Pietro D. Lampos V. (2016). Predicting judicial decisions of the European court of human rights: a natural language processing perspective. PeerJ Comput. Sci. 2:e93. 10.7717/peerj-cs.93

[B4] Allen L. E. Engholm C. R. (1979). The need for clear structure in plain language legal drafting. U. Mich. JL Reform 13:455.

[B5] Bartolini C. Lenzini G. Santos C. (2018). “An agile approach to validate a formal representation of the GDPR,” in JSAI-isAI Workshops. p. 160–176.

[B6] Barwise J. (1977). “An introduction to first-order logic,” in Studies in Logic and the Foundations of Mathematics, Vol. 90 (Elsevier), 5–46. 10.1016/S0049-237X(08)71097-8

[B7] Benzmüller C. (2019). Universal (meta-) logical reasoning: recent successes. Sci. Comput. Prog. 172, 48–62. 10.1016/j.scico.2018.10.00831016212

[B8] Burley A.-M. Mattli W. (1993). Europe before the court: a political theory of legal integration. Int. Organ. 47, 41–76. 10.1017/S0020818300004707

[B9] Church A. (1940). A formulation of the simple theory of types. J. Symbol. Logic 5, 56–68. 10.2307/2266170

[B10] Clark K. L. (1978). “Negation as failure,” in Logic and Data Bases (Springer), 293–322. 10.1007/978-1-4684-3384-5_11

[B11] Colmerauer A. (1990). “An introduction to prolog III,” in Computational Logic (Springer), 37–79. 10.1007/978-3-642-76274-1_2

[B12] Connell N. (1987). Expert systems in accountancy: a review of some recent applications. Account. Bus. Res. 17, 221–233. 10.1080/00014788.1987.9729802

[B13] Dastani M. Dong H. van der Torre L. (2020). “Logic and argumentation,” in Third International Conference, CLAR 2020, Hangzhou, China, Proceedings. Lecture Notes in Computer Science 12061 (Springer).

[B14] Durkin J. (1996). Expert systems: a view of the field. IEEE Intell. Syst. 11, 56–63. 10.1109/64.491282

[B15] Edelman L. B. (1992). Legal ambiguity and symbolic structures: organizational mediation of civil rights law. Am. J. Sociol. 97, 1531–1576. 10.1086/229939

[B16] Francesconi C. Borges G. Sorge C. (2022). “Legal knowledge and information systems,” in JURIX 2022: The Thirty-fifth Annual Conference, Saarbrücken, Germany, 14-16 December 2022. Frontiers in Artificial Intelligence and Applications, Vol. 362 (IOS Press).

[B17] Fungwacharakorn W. Tsushima K. Satoh K. (2021). Resolving counterintuitive consequences in law using legal debugging. Artif. Intell. Law 29, 541–557. 10.1007/s10506-021-09283-7

[B18] Hashmi M. Governatori G. (2018). Norms modeling constructs of business process compliance management frameworks: a conceptual evaluation. Artif. Intell. Law 26, 251–305. 10.1007/s10506-017-9215-8

[B19] Heimtz F. Milano M. O'Sulivan B. (2020). “Trustworthy AI - Integrating Learning, Optimization and Reasoning,” in First International Workshop, TAILOR 2020, Virtual Event, September 4-5, 2020, Revised Selected Papers. Lecture Notes in Computer Science 12641 (Springer). 10.1007/978-3-030-73959-1

[B20] Hughes J. (1995). “The design of a pretty-printing library,” in International School on Advanced Functional Programming eds, Jeuring J Meijer E: (Båstad: Springer), 53–96. 10.1007/3-540-59451-5_3

[B21] Huttner L. Merigoux D. (2022). Catala: moving towards the future of legal expert systems. Artif. Intell. Law 1–24. 10.1007/s10506-022-09328-5

[B22] Kornilov V. Glushan V. Lozovoy A. Y. (2021). “Metric for evaluation of machine translation quality on the bases of edit distances and reverse translation,” in 2021 IEEE 15th International Conference on Application of Information and Communication Technologies (AICT), 1–6. 10.1109/AICT52784.2021.9620304

[B23] Kowalski R. (2020). “Logical English,” in Proceedings of Logic and Practice of Programming (LPOP).

[B24] Kowalski R. Datoo A. (2022). Logical English meets legal English for swaps and derivatives. Artif. Intell. Law 30, 163–197. 10.1007/s10506-021-09295-3

[B25] Langley C. Spenser C. (2007). Visirule Tutorial.

[B26] Leith P. (1986). Fundamental errors in legal logic programming. Comput. J. 29, 545–552. 10.1093/comjnl/29.6.545

[B27] Libal T. Pascucci M. (2019). Proceedings of the Seventeenth International Conference on Artificial Intelligence and Law, ICAIL 2019, (Montreal, QC, Canada), p. 63–72. 10.1145/3322640.33267

[B28] McGraw K. L. Harbison-Briggs K. (1989). Knowledge Acquisition: Principles and Guidelines. Prentice-Hall, Inc.

[B29] Mernik M. Heering J. Sloane A. M. (2005). When and how to develop domain-specific languages. ACM Comput. Surveys 37, 316–344. 10.1145/1118890.1118892

[B30] Meyer B. (2008). Seven principles of software testing. Computer 41, 99–101. 10.1109/MC.2008.306

[B31] Mills M. (2016). Artificial Intelligence in Law: The State of Play 2016. Thomson Reuters Legal executive Institute.

[B32] Mockus M. Palmirani M. (2017). “Legal ontology for open government data mashups,” in 2017 Conference for E-Democracy and Open Government (CeDEM) (Krems), 113–124. 10.1109/CeDEM.2017.25

[B33] Morris J. (2021). “Constraint answer set programming as a tool to improve legislative drafting: a rules as code experiment,” in Proceedings of the Eighteenth International Conference on Artificial Intelligence and Law (São Paulo Brazil), 262–263. 10.1145/3462757.3466084

[B34] Nipkow T. Wenzel M. Paulson L. C. (2002). Isabelle/HOL: A Proof Assistant for Higher-Order Logic. Springer. 10.1007/3-540-45949-9

[B35] Novotná T. Libal T. (2022). “An evaluation of methodologies for legal formalization,” in International Workshop on Explainable, Transparent Autonomous Agents and Multi-Agent Systems (Springer), 189–203. 10.1007/978-3-031-15565-9_12

[B36] Novotná T. Libal T. (2023). “Designing an experiment for comparing user interfaces for legal formalization,” in Presented at the International Workshop on Programming Languages and the Law on the 15 January.

[B37] Nute D. (2012). Defeasible Deontic Logic, Vol. 263. Springer Science & Business Media.

[B38] Palmirani M. Governatori G. (2018). “Modelling legal knowledge for GDPR compliance checking,” in JURIX, Vol. 313 (Groningen), 101–110.

[B39] Pierce B. C. (2002). Types and Programming Languages. MIT Press.

[B40] Prakken H. Sartor G. (2015). Law and logic: a review from an argumentation perspective. Artif. Intell. 227, 214–245. 10.1016/j.artint.2015.06.005

[B41] Robaldo L. Bartolini C. Palmirani M. Rossi A. Martoni M. Lenzini G. (2020). Formalizing GDPR provisions in reified i/o logic: the Dapreco knowledge base. J. Logic Lang. Inform. 29, 401–449. 10.1007/s10849-019-09309-z

[B42] Routen T. Bench-Capon T. (1991). Hierarchical formalizations. Int. J. Man Mach. Stud. 35, 69–93. 10.1016/S0020-7373(07)80008-3

[B43] Satoh K. Takahashi K. Kawasaki T. (2021). “Interactive system for arranging issues based on proleg in civil litigation,” in Proceedings of the Eighteenth International Conference on Artificial Intelligence and Law (São Paulo Brazil), 273–274. 10.1145/3462757.3466096

[B44] Schauer F. (2013). On the open texture of law. Grazer Philos. Stud. 87, 197–215. 10.1163/9789401210119_013

[B45] Sergot M. J. Sadri F. Kowalski R. A. Kriwaczek F. Hammond P. Cory H. T. (1986). The British nationality act as a logic program. Commun. ACM 29, 370–386. 10.1145/5689.5920

[B46] Soavi M. Zeni N. Mylopoulos J. Mich L. (2022). From legal contracts to formal specifications: a systematic literature review. SN Comput. Sci. 3, 1–25. 10.1007/s42979-022-01228-434723205

[B47] Steen A. Benzmüller C. (2018). “The higher-order prover leo-III,” in International Joint Conference on Automated Reasoning (Oxford: Springer), 108–116. 10.1007/978-3-319-94205-6_8 PMC6109767 30174358

[B48] Sulea O.-M. Zampieri M. Malmasi S. Vela M. Dinu L. P. Van Genabith J. (2017). Exploring the use of text classification in the legal domain. arXiv preprint arXiv:1710.09306.

[B49] Waterman D. A. (1985). A Guide to Expert Systems. Addison-Wesley Longman Publishing Co., Inc.

[B50] Weaver D. A. Bimber B. (2008). Finding news stories: a comparison of searches using Lexisnexis and Google news. J. Mass Commun. Q. 85, 515–530. 10.1177/107769900808500303

[B51] Zheng H. Xiong M. Verheij B. (2018). “Checking the validity of rule-based arguments grounded in cases: a computational approach,” in JURIX (Groningen), 220–224.

